# Evaluation of PhenoMATRIX® PLUS for the culture-based rectal screening of CPOs, ESBL-E, *Acinetobacter*, and VRE carriage

**DOI:** 10.3389/fmicb.2025.1624411

**Published:** 2025-07-04

**Authors:** Abdessalam Cherkaoui, Gesuele Renzi, Mireille Tittel-Elmer, Jacques Schrenzel

**Affiliations:** ^1^Bacteriology Laboratory, Division of Laboratory Medicine, Department of Diagnostics, Geneva University Hospitals, Geneva, Switzerland; ^2^Genomic Research Laboratory, Division of Infectious Diseases, Department of Medicine, Faculty of Medicine, Geneva University Hospitals, Geneva, Switzerland

**Keywords:** PhenoMATRIX® PLUS, CPOs, MDROs, ESBL-E, *Acinetobacter*, VRE, chromogenic media, WASPLab®

## Abstract

**Background:**

Multidrug-resistant organisms (MDROs) have become a serious public health concern worldwide. Screening for MDROs can encompass various phenotypic and genotypic approaches. Recently, culture-based MDROs screening has substantially benefitted from laboratory automation and artificial intelligence.

**Objectives:**

To evaluate the performance of PhenoMATRIX® PLUS for an institution-wide culture-based rectal screening for CPOs, ESBL-E, *Acinetobacter*, and VRE carriage.

**Methods:**

The number of non-duplicated rectal Eswabs specimens used to generate culture plate images for the PhenoMATRIX® PLUS machine learning phase amounted to 2′900 for chromID™ ESBL agar, 2′403 for chromID™ OXA-48 agar, 2′031 for CHROMagar™ *Acinetobacter* agar, and 9′333 for chromID™ VRE agar. All such plates and images were classified by manual reading as either positive or negative. The validation of the settings derived during the learning phase was achieved on an additional 4′517 non-duplicate specimens. Digital images of 22′585 media plates (i.e., five per specimen) were prospectively analyzed by PhenoMATRIX® PLUS. All media plates were incubated in the WASPLab® and imaged at predefined time points.

**Results:**

According to the manual work-up results, the output agreements of the PhenoMATRIX® PLUS for the three results “*no growth*,” “*negative*,” and “*Send to Reader*” reached 98.5, 99.6%. and 74.8%, respectively. Importantly, no false negative results were returned by PhenoMATRIX® PLUS in the present study.

**Conclusion:**

PhenoMATRIX® PLUS contributes to a significant reduction in the workload. Therefore, technologists’ routine tasks have evolved to the confirmatory tests of the presumptively positive colonies, allowing better management of the laboratory staff and the resources.

## Introduction

Over the last decade, multidrug-resistant organisms (MDROs) have become a serious public health concern worldwide. Public health agencies have targeted various MDROs requiring enhanced barrier precautions in the hospital setting (Sartelli et al., 2024). This is especially relevant for carbapenemase-producing organisms (CPOs), *Acinetobacter* spp., vancomycin-resistant Enterococci (VRE), and extended-spectrum β-lactamase-producing Enterobacterales (ESBL-E). Swift and accurate detection of these MDROs is of paramount importance, since their patient-to-patient transmission is facilitated by their location on mobile genetic elements and the surrounding high antibiotic selective pressure (Mancuso et al., 2023). Detection of carriers triggers infection control procedures at an early stage to prevent the spread of these MDROs within the hospital environment, besides the enforcement of appropriate antibiotic stewardship measures (Durazzi et al., 2023).

Defining a workflow providing simple, rapid, reliable, and affordable routine diagnostic methods to identify carriers of such MDROs constitutes a challenge. Nowadays, several molecular assays including PCR panels or loop-mediated isothermal amplification (LAMP) assays are available (Tenover, 2021; Skally et al., 2025). These assays can be used for the screening, directly from samples (Novazzi et al., 2024). However, their analytical performances vary considerably, not to mention their costs (Kamel et al., 2022). Today, the most widespread approach for the screening of MDROs carriers is typically a combination of molecular assays coupled to culture and phenotypic tests (Dortet et al., 2016; Ciesielczuk et al., 2018; Gelmez et al., 2021).

The implementation of chromogenic culture media has considerably improved the performance of screening by cultures (Birlutiu et al., 2025). In addition, culture testing has largely benefitted from laboratory automation and the routine usage of artificial intelligence (AI). PhenoMATRIX® PLUS (Copan, Brescia, Italy) is a software designed to streamline culture-based workflows. It can sort and process culture media plates according to predefined rules. PhenoMATRIX® PLUS enables the automatic release of the negative results to the electronic patients’ file and the automatic discharge of the negative plates from the incubators.

The main objective of the present study is to assess the performance of PhenoMATRIX® PLUS for an institution-wide culture-based rectal screening to detect CPOs, ESBL-E, *Acinetobacter*, and VRE carriage.

## Materials and methods

### Setting

Geneva University Hospitals are a Swiss tertiary care center. In 2024, the bacteriology laboratory processed 196′456 specimens, for a total of 423′614 analyses. The screening for carbapenemase-producing organisms (CPOs), extended-spectrum β-lactamase-producing Enterobacterales (ESBL-E), *Acinetobacter*, and vancomycin-resistant *Enterococcus* (VRE) carriers was carried out on 13′728 rectal Eswabs (7.0% of our activity, in volume). For the screening of MRSA carriers, we processed 17′407 nasal and inguinal/perineal Eswabs (4.1% of our activity). Current hours of operation of the laboratory span from 7: 30 A.M. to 7: 30 P.M. Monday to Friday; Saturday from 7: 30 A.M. to 5: 00 P.M.; and Sunday from 7: 30 A.M. to 1: 00 P.M.

### Culture-based rectal screening of CPOs, ESBL-E, *Acinetobacter*, and VRE

Rectal CPOs, ESBL-E, *Acinetobacter*, and VRE screening-ESwabs (490 CE.A, Copan Italia S.p.A. Brescia, Italy) were streaked by the WASP® (Copan) on chromID™ ESBL agar (bioMérieux), chromID™ OXA-48 agar, (bioMérieux), CHROMagar™ *Acinetobacter*, and chromID™ VRE agar (bioMérieux), in addition to a MacConkey agar (bioMérieux). This last medium was included to assess the quality of the sample. All media plates were incubated in the WASPLab® (Copan) and imaged at predefined time points. We applied the final incubation time points as previously validated for CPOs, ESBL-E and VRE (Cherkaoui et al., 2019; Cherkaoui et al., 2020). Digital images were manually read by the technologists. The identification of screened organisms and their antimicrobial susceptibility testing were performed during the day shift. For all presumptively positive CPOs, ESBL-E, and *Acinetobacter* colonies on chromogenic media, bacterial identification was performed by matrix-assisted laser desorption ionization–time of flight mass spectrometry (MALDI-TOF/MS) (MBT Compass 4.1, library version 11.0 (11′410 spectra), Bruker Daltonics, Bremen, Germany) according to the manufacturer’s instructions. The antimicrobial susceptibility testing (AST) was carried out by disk diffusion according to EUCAST recommendations. The ESBL-E was confirmed phenotypically by double-disc synergy tests (DDST20 and DDST30) using Mueller Hinton E agar (MHE) (bioMérieux).

We used the eazyplex® SuperBug complete tests (Amplex Biosystems GmbH, Giessen, Germany) to identify the genes encoding carbapenemases in isolates with reduced susceptibility to carbapenems according to the EUCAST screening breakpoints. This system is based on a loop-mediated isothermal amplification. The carbapenemases detected by the eazyplex® SuperBug complete tests were KPC, NDM, VIM, OXA-23, OXA-40, OXA-48, OXA-58, and OXA-181. In addition, extended-spectrum β-lactamases from the CTX-M-1 and CTX-M-9 groups were also identified. Carbapenemase gene confirmation and sequencing were performed by the national reference center for early detection and monitoring of antibiotic resistance (NARA, Fribourg, Switzerland).

For all presumptively positive VRE colonies on chromID™ VRE agar, their identification by MALDI-TOF/MS and AST were followed by a qPCR assay targeting *vanA* and *vanB* (Thermo Fisher Scientific™—QuantStudio™ 5 Real-Time PCR and Absolute™ qPCR MasterMIX Thermo Scientific ABgene®) (Naserpour Farivar et al., 2014).

### PhenoMATRIX® PLUS: machine learning phase

The number of non-duplicated rectal Eswabs specimens used to generate culture plate images amounted to 2′900 for chromID™ ESBL agar, 2′403 for chromID™ OXA-48 agar, 2′031 for CHROMagar™ *Acinetobacter* agar, and 9′333 for chromID™ VRE agar. All such plates and images were classified by manual reading as either positive or negative. These images were then used as the ground truth to train the convolutional neural networks.

### PhenoMATRIX® PLUS: validation phase

The validation of the settings derived during the learning phase was conducted on an additional 4′517 non-duplicate specimens assigned to our laboratory for analysis. Digital images of 22′585 media plates (i.e., five per specimen) were prospectively analyzed by PhenoMATRIX® PLUS. Seeking maximum accord, all the media plate images were read manually by the technologists, but they were kept blind of the results dispensed by PhenoMATRIX® PLUS. Discrepancies between PhenoMATRIX® PLUS and manual reading were recorded and reviewed by an independent reader.

**Figure 1:**
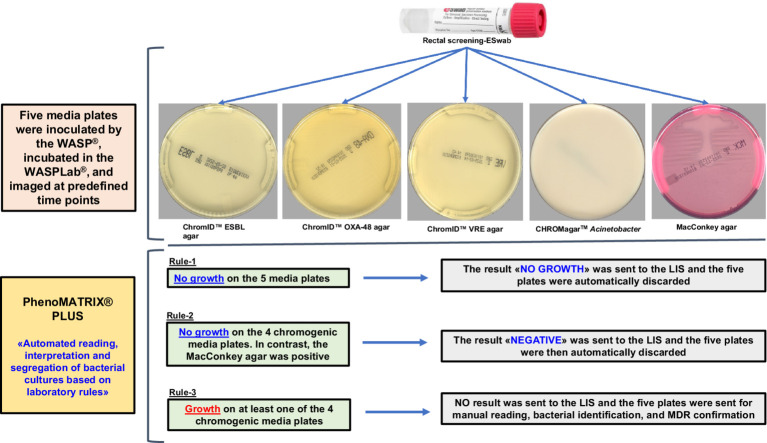
Laboratory screening workflow for CPOs, ESBL-E, *Acinetobacter* spp., and VRE.

Figure 1 depicts the laboratory screening workflow for carbapenemase-producing organisms (CPOs), extended-spectrum β-lactamase-producing Enterobacterales (ESBL-E), *Acinetobacter* spp., vancomycin-resistant Enterococci (VRE). Rule-2 was applied to remove the bias associated with the non-specific bacterial growth on MacConkey agar. Therefore, all the “no growth” and “negative” results defined by PhenoMATRIX® PLUS were only associated to the specific growth on chromogenic media used in this study.

## Results

### PhenoMATRIX® PLUS performance for the rectal screening of CPOs, ESBL-E, *Acinetobacter*, and VRE

**Figure 2:**
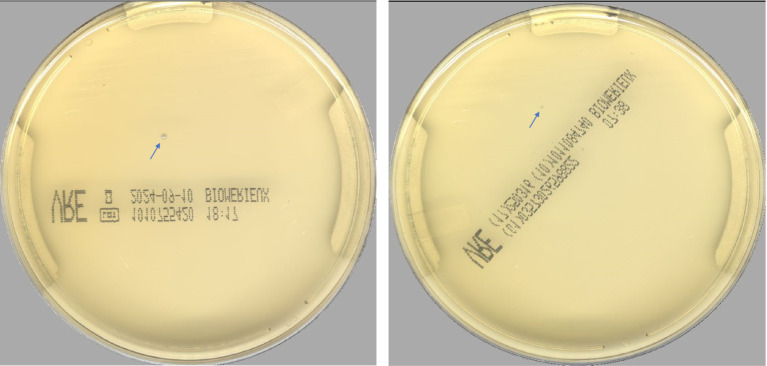
Unspecific colonies on the ChromID™ VRE agar.

**Figure 3:**
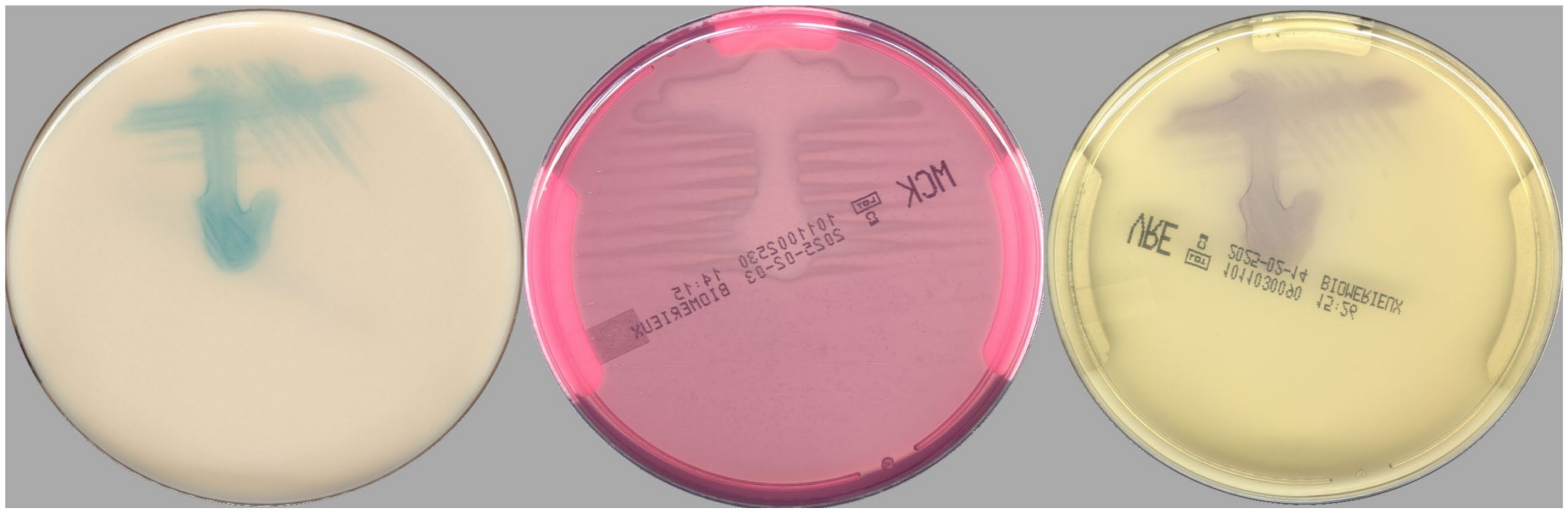
Images of culture media plates with no bacterial growth but only sample traces.

**Figure 4:**
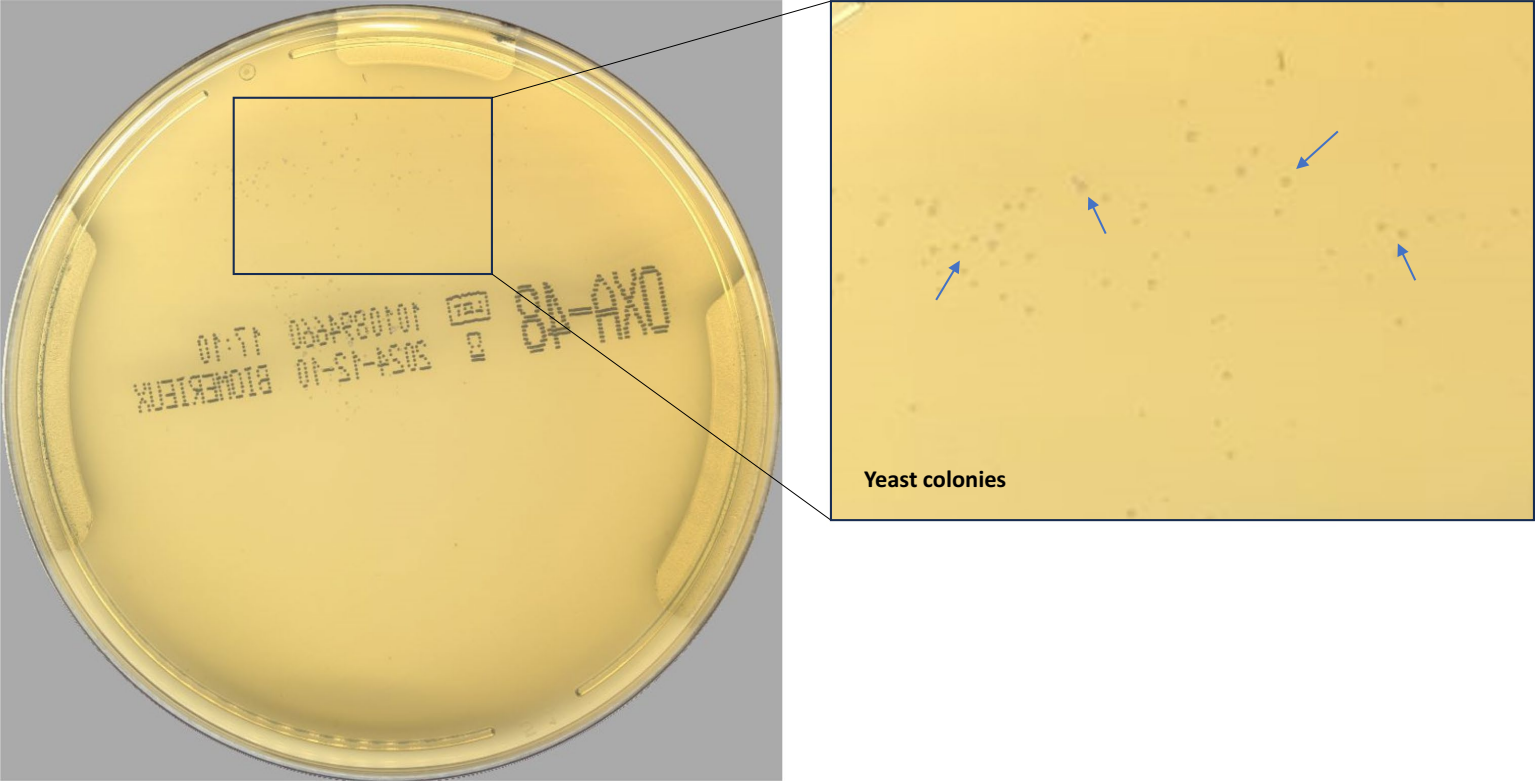
Yeast colonies on chromID™ OXA-48 agar plate.

According to the PhenoMATRIX® PLUS workflow depicted in Figure 1, the clinical performances of PhenoMATRIX® PLUS for the screening of CPOs, ESBL-E, *Acinetobacter*, and VRE were compared to the manual work-up results (Table 1). The results were broken down by media plates (Table 1a) and samples (Table 1b). Among the 4′517 non-duplicated rectal Eswabs specimens analyzed, six were defined by PhenoMATRIX® PLUS as “*No growth*” but reported as “*Negative*” by manual reading. The image review of these 30 media plates showed that discordance could be explained by the presence of a few unspecific colonies on the chromID™ VRE agar (Figure 2). Seven specimens were segregated by PhenoMATRIX® PLUS as “*Negative*” but reported as “*No growth*” by manual reading. There was no evidence of bacterial growth on the MacConkey agar plates but only sample traces according to the image review of the media (Figure 3). Forty specimens were segregated by PhenoMATRIX® PLUS as “*Send to Reader*” but reported as “*No growth*” by manual reading. No evidence of bacterial growth was revealed but there were only sample traces (Figure 3). Finally, 554 specimens were segregated by PhenoMATRIX® PLUS as “*Send to Reader*” but reported as “*negative*” by manual reading. Yeast colonies were observed on some chromID™ OXA-48 agar plates (Figure 4). Growth of small colonies of Gram-positive bacteria on chromID™ OXA-48 agar and unspecific colonies on chromID™ VRE agar, explained discordance for most of these specimens. According to the manual work-up results, the output agreements of the PhenoMATRIX® PLUS for the three results “*no growth*,” “*negative*,” and “*Send to Reader*” reached 98.5, 99.6%, and 74.8%, respectively.

**Table 1:** Clinical performances of PhenoMATRIX® PLUS for the screening of CPOs, ESBL-E, *Acinetobacter*, and VRE compared to the manual work-up results.

(a)	Manual work-up (culture media plates)						
		No growth	Negative	Send to reader	Total		Output agreement
PhenoMATRIX® PLUS	No growth	1945	30	0	1975	8.7%	98.5%
	Negative	35	8,780	0	8,815	39.0%	99.6%
	Send to Reader	200	2,770	8,825	11,795	52.2%	74.8%
	Total	2,180	11,580	8,825	22,585	100.0%	
		9.7%	51.3%	39.1%	100.0%		
(b)	Manual work-up (sample)						
		No growth	Negative	Send to reader	Total		Output agreement
PhenoMATRIX® PLUS	No growth	389	6	0	395	8.7%	98.5%
	Negative	7	1756	0	1763	39.0%	99.6%
	Send to Reader	40	554	1765	2,359	52.2%	74.8%
	Total	436	2,316	1765	4,517	100.0%	
		9.7%	51.3%	39.1%	100.0%		

The results were broken down by culture media plates (a) and samples (b).

## Discussion

PhenoMATRIX® PLUS enables complete handling of the negative specimens (i.e., assessment of the culture images, automatic release of the results to the LIS, and discarding of the negative plates without technical validation). The accuracy of such software has been previously evaluated for MRSA screening (Cherkaoui et al., 2024). In the present study, we defined a culture-based protocol for rectal Eswab CPOs, ESBL-E, *Acinetobacter*, and VRE screening. This protocol included four chromogenic media and one MacConkey agar plate, the latter for sampling quality. The settings used for PhenoMATRIX® PLUS during the machine learning phase were not based on the specific color of the chromogenic media but only on the organisms’ growth since no specific color is defined by the manufacturers of CPOs and ESBL-E. Thus, three predefined rules were applied in the validation phase of the PhenoMATRIX® PLUS «*No growth*», «*Negative*», and «*Send to Reader*» as explained above. PhenoMATRIX® PLUS ensured the total and independent management of 48% (10′790/22′585) of the media plates flagged as «*No growth*» or «*Negative*» which corresponded to 2′158 negative specimens. The remaining media plates (52%, 11′795/22′585) whose organisms’ growth was segregated as presumptive positive cases still required confirmation by the technologists. In about 8% of the media plates analyzed, PhenoMATRIX® PLUS declared them as positive without evidence of bacterial growth but only due to sample traces. These false positive results should be the subject for upcoming improvement of the PhenoMATRIX® PLUS algorithm. Importantly, no false negative results were returned by PhenoMATRIX® PLUS in the present study.

Currently, PhenoMATRIX® PLUS is coupled to another software (Valab® expert system, Flourens, France) in our lab workflow. After results are automatically sent to the LIS by PhenoMATRIX® PLUS, Valab® automatically validates them and updates immediately the electronic patient file.

The present study has some limitations: (i) PhenoMATRIX® PLUS settings for the chromID™ VRE agar were based on bacterial growth and not on the specific color of the colonies. Settings based on the specific color on VRE chromogenic medium could have reduced the number of false positives, and (ii) sample traces were considered in several cases as bacterial growth by PhenoMATRIX® PLUS.

## Conclusion

Facing increasing numbers of specimens dedicated to the screening of MDROs carriage, the implementation of PhenoMATRIX® PLUS contributes to a significant reduction in workload. Therefore, technologists’ routine tasks have evolved to the confirmatory tests of the presumptively positive colonies, allowing better management of the laboratory staff and resources. Some adjustments appear necessary to improve the specificity of PhenoMATRIX® PLUS for the rectal screening of MDROs, but the advantages of this software in terms of sensitivity and workload reduction in the routine lab are already obvious.

## Data Availability

The raw data supporting the conclusions of this article will be made available by the authors, without undue reservation.
